# Atypical presentation of herpes simplex virus type 1 infection in paediatric burns patients in a large tertiary hospital, South Africa

**DOI:** 10.4102/ajlm.v8i1.916

**Published:** 2019-10-23

**Authors:** Mpho L. Sikhosana, Asma Salloo, Monica Birkhead, Kerrigan McCarthy

**Affiliations:** 1 South African Field Epidemiology Training Programme, National Institute for Communicable Diseases, Division of National Health Laboratory Services, Johannesburg, South Africa; 2 Department of Critical Care, Chris Hani Baragwanath Academic Hospital, Soweto, South Africa; 3 Department of Critical Care, University of Witwatersrand, Johannesburg, South Africa; 4 Centre for Emerging Zoonotic and Parasitic Diseases, National Institute for Communicable Diseases, Division of National Health Laboratory Services, Johannesburg, South Africa; 5 Division for Public Health Surveillance and Response, National Institute for Communicable Diseases, Division of National Health Laboratory Services, Johannesburg, South Africa

**Keywords:** herpes simplex virus type 1, burns, paediatrics, South Africa

## Abstract

**Introduction:**

Herpes simplex virus has been reported in the literature to commonly complicate burn wounds. However, there is paucity of such data in the South African setting.

**Case presentation:**

Eight paediatric burns patients with ages ranging between 10 months and 5 years presented with a febrile maculopapular rash illness in a paediatric ward of a large South African tertiary hospital. The rash became vesicular in three cases, involving the limbs and face. Varicella was suspected.

**Management and outcome:**

Medical records of suspected cases were reviewed. Blood, vesicular fluid and scab samples were collected. Electron microscopy of vesicular fluid revealed herpes virus particles. Laboratory testing confirmed herpes simplex virus type 1.

**Conclusion:**

Herpes simplex virus type 1 infection can present atypically in burns patients.

## Introduction

Prevention of infection is an important part of managing burns patients. Prophylactic administration of antibiotics or antivirals is not routine, with treatment warranted only in patients in whom infection is highly suspected or proven by laboratory testing.^1,2,3,4^ Differences in the clinical presentation of skin infections in burns patients are not always apparent; however, infectious and non-infectious causes must be included in the differential diagnosis.^1,5^

Viral infections by members of the Herpesviridae family, including herpes simplex virus type 1 (HSV1), cytomegalovirus and varicella-zoster virus, have been found to occur commonly in severely burnt patients.^4,5,6^ These infections can either be primary or due to reactivation of a latent virus. Over 3700 million people between 0 and 49 years have been estimated to be latently infected with HSV1, with Africa being one of the most affected regions globally.^7^ As such, of all the herpesviruses, HSV1 is the most frequently reported virus that complicates burns, whereas varicella-zoster virus infections occur rarely.^4^ Herpes simplex virus type 1 infections present as a febrile illness 1 to 3 weeks following extensive, full-thickness burns injuries.^5,6^ The infection frequently occurs in patients with burns to the head or neck. When associated with burn wounds, the lesions typically begin as clustered vesicles or vesicular pustules within or around the wound margins, with subsequent impaired wound healing.^5,6^ Herpes simplex virus type 1 lesions can resemble those of pox viruses, with the latter also having been identified in burns patients.^8,9^ Cytomegalovirus infections have not been shown to cause severe complications or increase mortality in burns patients.^6^ However, the presence of both primary and reactivation cytomegalovirus infections in severely burned children has been recorded previously.^6^ Underlying herpes viral infections can promote bacterial infections, resulting in prolonged hospitalisation, need for mechanical ventilation, delayed recovery and higher mortality rates.^1,4,10,11^

Herpes viral infections in burns patients have not been described in the South African setting. However, due to the contagious nature of these infections, there are implications for infection prevention and control practices, particularly in the sub-population of immunosuppressed burns patients. In addition, possible complications such as HSV1-associated encephalitis make knowledge on the management of these infections important.

## Ethical considerations

Due to the initial presentation as a febrile maculopapular rash illness, the cluster was initially investigated as a possible measles outbreak. All outbreak investigations, which would include history taking (in this case, from the parents in light of the patients’ ages), patient examination as well as sample collection, that are conducted by the National Institute for Communicable Diseases have ethics clearance from the Human Research Ethics Committee of the University of the Witwatersrand, South Africa (M160667, 2016–2020). In terms of this ethics clearance, patient consent is not required and any patient specimen collected is anonymised

## Case presentation

**FIGURE 1:**
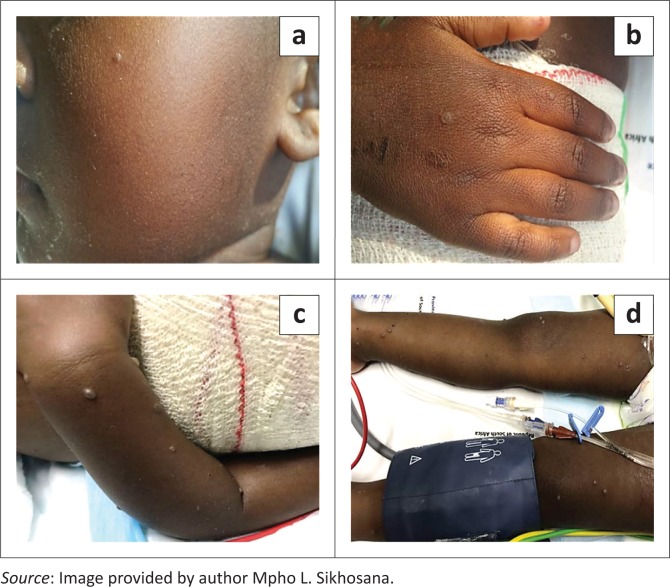
Vesicular lesions on two of the cases, Gauteng, South Africa, July 2017. (a, b) Vesicular lesions seen on one of the cases that initially presented with a maculopapular rash. (c, d) Vesicular lesions on the third case. This patient was not part of the initial cluster with a maculopapular rash.

During July 2017, seven paediatric burns patients between the ages of 10 months and 5 years presented with a maculopapular rash at a tertiary hospital in Gauteng, South Africa. Four of these patients were female. The rash was associated with both fever and coryza in four of the cases. The characteristics of the cases involved in this cluster are shown in Table 1. The cluster was reported to the National Institute for Communicable Diseases. Due to a concurrent measles outbreak in the province, measles was initially suspected. The rash subsequently evolved and became vesicular in two of the cases, affecting the limbs and hands in one of the cases (Figure 1). Contemporaneously, an eighth patient presented with a vesicular rash on the trunk and on both upper and lower extremities bilaterally. Of note is that this patient did not initially present with a maculopapular rash. Varicella-zoster became a differential diagnosis. As children are not routinely immunised against varicella in South Africa’s public health sector, the cost, availability and resource utilisation of prophylactic varicella immunoglobulins for the cases posed a number of challenges.^6^ The natural history in the cases that developed the vesicular lesions was also atypical of the varicella-zoster infection. There was therefore a possibility of administering the immunoglobulins unnecessarily. Other possible diagnoses that were considered included enterovirus and pox infections. An investigation was initiated by the National Institute for Communicable Diseases in order to establish the cause of the illness. A composite case definition that was used to identify other cases in the ward included any patient who was admitted to the unit during July 2017, presenting with a maculopapular or vesicular rash, with or without fever or coryza. The medical records of suspected cases were reviewed using a pre-designed case investigation form. Cases were also examined for any residual or new symptoms. Further information on the history of the illness was also obtained from the caregivers of some of the cases. Blood samples were collected for serological and molecular testing using the following assays: Siemens Enzygnost® measles IgM and rubella IgM EIA (Siemens, Marburg, Germany), Liaison® VZV IgM CLIA (DiaSorin, Saluggia, Italy), HerpeSelect® HSV-1 EIA (Focus Diagnostics, Cypress, California, United States), Genesig® Herpes Simplex Virus type 1 and 2 real-time polymerase chain reaction kit (PrimerDesign Ltd, Southampton, Hampshire, United Kingdom). Vesicular fluid and scab samples were collected and submitted to the National Institute for Communicable Diseases for transmission electron microscopy using negative staining.^9^

**TABLE 1:** Characteristics of the cases in the febrile rash illness at the tertiary hospital in Gauteng, South Africa, July 2017.

Case	Age	Sex	% burn	Burn site	Maculopapular rash	Vesiccular rash	Fever	Coryza	Results				Final diagnosis
									Measles	Rubella	HSV1†	EM	
1	2 years	F	8.0%	Face	No	Yes	Unknown	Unknown	-	-	positive	positive	Confirmed HSV1‡
2	3 years	F	18.0%	-	Yes	Yes	No	-	-	-	positive	positive	Confirmed HSV1‡
3	2 years	F	3.0%	-	Yes	Yes	Unknown	No	negative	negative	positive	-	Confirmed HSV1‡
4	1 years	M	11.0%	Face	Yes	Yes	Yes	No	negative	negative	-	-	Probable HSV1§
5	9 years	M	20.0%	-	No	Yes	Yes	Unknown	-	-	-	-	Probable HSV1§
6	2 years	M	13.0%	-	Yes	No	Yes	Yes	negative	negative	-	-	Non HSV case
7	4 years	F	5.0%	-	Yes	No	Yes	Yes	negative	negative	-	-	Non HSV case
8	1 years	M	9.0%	-	Yes	No	Yes	Yes	-	-	-	-	Non HSV case
9	10 months	F	1.5%	-	Yes	No	Yes	No	-	-	-	-	Non HSV case
10	5 years	M	20.0%	-	Yes	No	Yes	Yes	negative	negative	-	-	Non HSV case

F, female; M, male; HSV, herpes simplex virus; EM, electron microscopy.

† , Herpes simplex virus1 polymerase chain reaction.

‡ , Laboratory-confirmed HSV1.

§ , Two probable HSV1 cases with a documented vesicular rash identified from the medical record review, but from whom no clinical specimens were obtained for laboratory confirmation.

**FIGURE 2:**
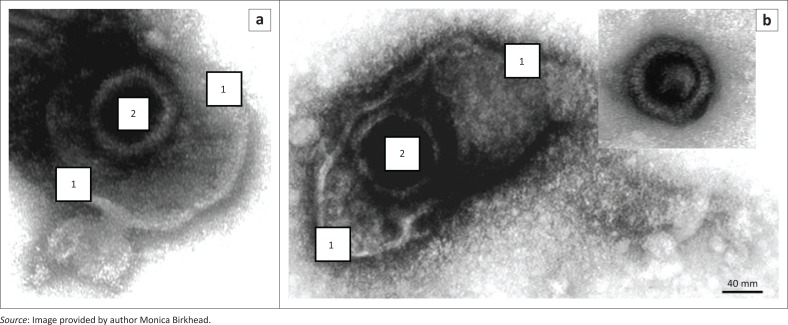
Transmission electron microscopy of negatively-stained virions in vesicular fluid, Gauteng, South Africa, July 2017. (a) and (b) show typical Herpesviridae particles with an icosahedral nucleocapsid (2) surrounded by a loose envelope (1). During collection and processing of vesicular fluids, the fragile envelope frequently ruptures to release the nucleocapsid, which can be seen to be composed of characteristic cylindrical capsomers (inset b).

**FIGURE 3:**
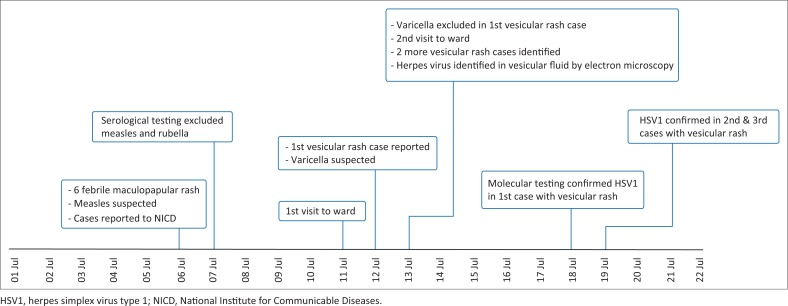
Sequence of events during the investigation, Gauteng, South Africa, July 2017.

Measles, rubella and varicella infections were excluded based on negative serological testing. Viral particles consistent with those of the Herpesviridae family were seen on electron microscopy of the vesicular fluid, while no virions were detected in the scab specimen (Figure 2). Based on these electron microscopy findings, further laboratory testing for herpes viruses was conducted, and HSV1 was confirmed based on serological and molecular testing. The medical record review of other patients in the ward identified two other cases with a documented vesicular rash. A timeline showing the sequence of events in this investigation is shown in Figure 3.

## Management and outcomes

Due to the initial differential diagnosis of measles, cases were given vitamin A, and measles vaccine was administered to all ward contacts below the age of 5 years. All of the cases recovered completely without any complications.

## Discussion

The presentation of HSV1 infection in this cluster was atypical for several reasons. Firstly, HSV infections are usually associated with more extensive burns.^4,5,6^ All of the cases in this cluster had partial thickness burn wounds that were ≤ 20% of the total body surface area. Secondly, unlike the commonly described distribution of the vesicular rash within or around the wound margins, the lesions occurred peripherally from the burn wounds in all except one of the cases. The vesicular rash was also distributed on the limbs and torso in some of the cases, with only one case having orolabial involvement.

The fact that no virions were observed in the scab using transmission electron microscopy is in keeping with the presentation of herpes viruses. This finding also aided in the exclusion of pox viruses, as the latter are typically found in the crusted lesions. Pox viruses are also more robust and long-lived compared to herpes viruses which have more delicate and pleomorphic envelopes.^9^

The incidence of HSV1 infections have been estimated to occur more commonly in the 0–4 year age group in both boys and girls in Africa.^7^ Due to the spatial and temporal association of the cases, primary nosocomial infection is the likely mode of transmission. However, due to a high HSV1 seroprevalence in our study setting, the reactivation of latent HSV1 is also a likely possibility.

HSV is transmitted through contact with vesicular lesions, and this was the most likely mode of transmission in this cluster. This highlights the importance of adhering to standard precautions, particularly in burns patients in whom the protective skin barrier has been compromised. We postulate that the communal dressing room, where most of the cases had their wounds dressed prior to the onset of the illness, was most likely where transmission occurred.

### Limitations

Limitations of this outbreak investigation included delayed identification of two probable cases found on the medical records review. As their vesicular lesions had already healed, appropriate samples were not available for laboratory confirmation of HSV1. In addition, comparative sequencing in the three cases with laboratory-confirmed HSV1 could not be conducted due to unavailability of residual specimens.

### Conclusion

Although HSV1 infection in burns patients has been described in other international settings, a literature review showed that the illness has not been well described in the South African setting. Treatment of laboratory-confirmed HSV infection includes intravenous acyclovir at 5–10 mg/kg 8 hourly for 10–14 days, with or without the addition of topical acyclovir.^4,5,6^ This management was not prescribed for the cases in this cluster, but there were subsequently no complications in any of the patients. Prevention of the nosocomial spread of HSV1 includes isolating infected patients and avoiding the use of communal facilities by cases until the vesicular lesions have healed. Surgery to infected wounds should also be deferred until vesicles have resolved.
